# Review of Automated Insulin Delivery Systems for Type 1 Diabetes and Associated Time in Range Outcomes

**DOI:** 10.17925/EE.2022.18.1.27

**Published:** 2022-05-20

**Authors:** Armaan Nallicheri, Katherine M Mahoney, Hanna A Gutow, Natalie Bellini, Diana Isaacs

**Affiliations:** 1. Close Concerns, San Francisco, CA, USA; 2. R&B Medical Group, Williamsville, NY, USA; 3. Cleveland Clinic, Cleveland, OH, USA

**Keywords:** Automated insulin delivery, type 1 diabetes mellitus, time in range, glycemic management, dual hormone pump, continuous glucose monitoring, hybrid closed loop system

## Abstract

Automated insulin delivery (AID) systems play an important role in the management of type 1 diabetes mellitus (T1DM). These systems include three components: a continuous glucose monitor (CGM), an insulin pump and an algorithm that adjusts the pump based on the CGM sensor glucose readings. They are not fully automated and still require the user to administer bolus insulin doses for food. Some AID systems have automatic correction boluses, while others only have automatic basal or background insulin adjustments. As CGM has become more accurate and the technology has evolved, AID systems have demonstrated improved glycaemic outcomes. The clinical evaluation of AID systems in randomized controlled trials and real-world studies have shown their utility in helping glycaemic management. In this review, we compare AID systems that are commercially available in the US and summarize the literature, with a special focus on time in range in T1DM. The review also discusses new AID systems on the horizon and explores considerations for personalized care.

Automated insulin delivery (AID) systems play an important role in the management of type 1 diabetes mellitus (T1DM) by helping users achieve the recommended glucose targets while reducing the episodes of hypoglycemia.^1^ First-generation AID systems, including MiniMed^™^ 630G (Medtronic, Dublin, Ireland) and Basal-IQ (Tandem Diabetes Care, Inc., San Diego, CA, USA) featured prespecified glucose boundaries at which to suspend insulin pump delivery to prevent hypoglycaemia; in contrast, second-generation systems focused on “hybrid closed-loop” technology, which automatically delivers basal insulin based on sensor data while incorporating user-initiated mealtime boluses. Current systems in development aim to eliminate the need for meal announcements and, eventually, to fully “close the loop” by adding dual hormone capabilities that regulate blood glucose without user input.^2^

The clinical evaluation of these systems through randomized controlled trials and real-world studies has demonstrated their utility in helping glycemic management.^3–17^ Traditional AID systems have three components:

- an insulin pump
- a continuous glucose monitor (CGM)
- an algorithm that titrates insulin by parsing sensor glucose levels.

While most commercially available and pipeline AID systems have similar components, they also have important feature differences. For example, some have automatic correction boluses, such as Control-IQ Technology^™^ (Tandem Diabetes Care, Inc., San Diego, CA, USA) and MiniMed™ 780G (Medtronic, Dublin, Ireland), while others offer varying degrees of smartphone control (e.g., Control-IQ; Omnipod^®^ 5, Insulet, Billerica, MA, USA).^18^ In this review, we highlight relevant outcomes from key research studies for five prominent AID systems while assessing the strengths and limitations of each system. We emphasize time in range (TIR), defined as the percentage of time spent between 70 and 180 mg/dl, other CGM-derived outcomes, and glycated haemoglobin A1c (HbA1c) as key metrics to assess the efficacy of the AID systems.

## Evaluation of automated insulin delivery systems

### Medtronic's MiniMed 670G/770G

In 2017, MiniMed^™^ 670G (Medtronic, Dublin, Ireland) was the first AID system to be made commercially available. It comprises the Guardian Sensor^™^ 3 (Medtronic, Dublin, Ireland) CGM, the MiniMed^™^ 600 series pump (Medtronic, Dublin, Ireland) and a proportional integrated derivative control algorithm. The Guardian Sensor 3 must be calibrated two to four times a day and can last up to 7 days, although it is labelled adjunctively and thus cannot be used for insulin dosing without a confirmatory fingerstick. The system was formally discontinued earlier in 2021 after Medtronic announced its new MiniMed^™^ 770G (Medtronic, Dublin, Ireland) system, which uses the same MiniMed 670G algorithm with the updated MiniMed^™^ 780G pump hardware (series 700 pump). It continues to work with the Guardian 3 sensor.^19^

A non-randomized prospective pivotal trial, which led to the approval of the MiniMed 670G system, involved 94 adults and 30 adolescents and compared baseline data to 3 months on MiniMed 670G.^3^ There was a 5.0% improvement in TIR for adults and a 6.8% improvement in TIR for adolescents, which correspond to 1.2 and 1.7 additional hours in range per day on average, respectively. Adult and adolescent HbA1c decreased by 0.5% and 0.6%, respectively, compared with baseline. Moreover, adult and adolescent time spent <70 mg/dL was reduced by 3.0% (43 minutes) and 1.5% (22 minutes), respectively.

MiniMed 670G's paediatric pivotal trial showed similar TIR and HbA1c improvements to the adult pivotal trial.^4^ In a single-arm prospective trial studying 105 children with T1DM (age range: 7–13 years), participants witnessed a 8.8% improvement in TIR (from 56.2% to 65.0%) and an HbA1c drop of 0.4% (from 7.9% to 7.5%), in addition to approximately 30 minutes less spent <70 mg/dL (from 3.2% to 1.2%).^4^ Additional data, including real-world data, from key trials involving MiniMed 670G are presented in *Table 1*.^3–8^ The tables include TIR and time below range (<70 mg/dL). Time above range (over 180 mg/dL) can be calculated by subtracting TIR and time below range from 100%.

A 3-month real-world study involving 3,141 participants with T1DM who wore MiniMed 670G complements this robust set of clinical data.^8^ In this study, there was a 7% improvement in TIR (from 66% to 73%), translating to roughly 1.7 hours more TIR per day on average. While this study did not include HbA1c as a study outcome, the data demonstrate that MiniMed 670G improves TIR for users, while also slightly reducing time spent <70 mg/dL by 0.6% (from 2.7% to 2.1%; i.e. roughly 9 minutes per day).

### Medtronic's MiniMed 780G

Medtronic's next AID system is MiniMed 780G, which launched in Europe in 2020 and is under review by the US Food and Drug Administration (FDA).^20,21^ Marking a significant upgrade from the MiniMed 670G, the new system features automatic correction boluses and an adjustable target as low as 100 mg/dL. The series 700 pump with a proportional integrated derivative control algorithm also features Bluetooth connectivity, which will enable smartphone data transfer and promote cloud-sharing of pump data on remote patient monitoring platforms. In Europe, the system is now being sold with the Guardian Sensor^™^ 4 (Medtronic, Dublin, Ireland), which Medtronic has filed with the FDA as part of the MiniMed 780G submission package.^21^ The Guardian Sensor 4 would be Medtronic's first non-adjunctive CGM to be approved for fingerstick- and calibration-free use, marking a significant improvement over the Guardian Sensor 3. However, it will still be indicated for 7-days wear.^22^

Medtronic's MiniMed 780G was directly compared with the MiniMed 670G during the head-to-head randomized FLAIR study (Home use of MD-logic automated insulin delivery system: Safety and efficacy; ClinicalTrials.gov identifier: NCT03040414) in adolescents and young adults (n=113, ages 14–29) with T1DM.^7,23^ Participants were randomized to adopt either the MiniMed 670G or 780G, with crossover at 3 months. The Guardian Sensor 3 and MiniMed 670G pump were used in both arms to enable a direct comparison between the two groups. Although both the MiniMed 670G and 780G use resulted in improvements in TIR compared with baseline (57%), use of the MiniMed 780G resulted in a significantly greater improvement in TIR than use of the MiniMed 670G (6% versus 10%, or an increase in 1.4 hours per day versus an increase in 2.4 hours per day, respectively). HbA1c decreased from 7.9% at baseline to 7.6% in the MiniMed 670G arm and to 7.4% in the MiniMed 780G arm, which thus demonstrated a statistically significant change from baseline and between arms of the trial.

The pivotal trial results for the MiniMed 780G system were published in *Diabetes Technology & Therapeutics* and showed TIR improvements of 5.7% (from 68.8% to 74.5%) in adults and 11% (from 62.4% to 72.7%) in adolescents.^9^ To the overall group, the MiniMed 780G delivered an HbA1c reduction of 0.5% (baseline: 7.5%), and mean sensor glucose was reduced from 153 mg/dL to 148 mg/dL. Another study compared the MiniMed 780G with sensor-augmented pump (SAP) therapy and a predictive low glucose management (PLGM) algorithm (SAP + PLGM) in a randomized crossover trial.^10^ After enrolling 59 participants with T1DM (age range: 7–80 years), the study recorded an 11% improvement in TIR (from 59% to 70%), or an additional 2.6 hours per day in range, in participants in the MiniMed 780G arm, compared with a 1% decrease in TIR in the SAP + PLGM arm. There are few published randomized controlled trials or real-world data on the MiniMed 780G, but the available clinical studies involving this system are compiled in *Table 2*.^9–12^

### Tandem's Control IQ

Tandem's non-adjunctive t:slim X2^™^ pump with Control-IQ was the next AID system to launch in the USA in 2020 after the MiniMed 670G, and proved to be a very appealing option in many ways.^13^ Besides being able to receive software updates and being calibration free, the Control-IQ also allows users to program their basal rates and bolus doses for meals and corrections; therefore, this system represents a far more customizable and personalized approach to AID. In particular, the Control-IQ's exercise and sleep modes, as well as the FDA-cleared smartphone bolus functionality, offer users a unique advantage compared with Medtronic's AID systems.^14^ Tandem's Control-IQ system is composed of a Dexcom G6^®^ (DexCom, Inc., San Diego, CA, USA) CGM and TypeZero “treat to range” predictive control algorithm integrated into the interoperable t:slim X2 pump.^13^

During an adult pivotal trial, participants between 14 and 71 years old with T1DM who used Control-IQ witnessed a 10% improvement in TIR (from 61% to 71%) compared with those on SAP, who experienced no change from baseline.^13^ The mean HbA1c reduction in participants using Control-IQ was 0.3%, whereas HbA1c did not improve in the SAP arm. Furthermore, time spent <70 mg/dL improved by 2.0% (from 3.6% to 1.6%) in the Control-IQ arm versus 0.6% (from 2.9% to 2.3%) in the SAP arm – a difference of 29 minutes per day versus 9 minutes per day spent <70 mg/dL, respectively. A paediatric pivotal trial (age range: 6–13 years) also showed impressive improvements across all primary outcomes, with Control-IQ users experiencing a 14% improvement in TIR (from 53% to 67%) compared with the 4% improvement (from 51% to 55%) in SAP users.^15^ There was an HbA1c reduction of 0.6% in the Control-IQ group (from 7.6% to 7.0%), compared with a 0.3% reduction in the SAP group (from 7.9% to 7.6%).

In a year-long real-world retrospective study, the 9,451 participants (83% with T1DM; mean age: 43) saw a 10% improvement in TIR on average (from 63.6% to 73.6%), which corresponds to 2.4 additional hours per day spent in range.^24^ Time spent <70 mg/dL hovered around 1% on average for this cohort, both at baseline and after Control-IQ use. While there is no HbA1c data from this trial, the mean glucose management indicator at baseline was 7.3%. All studies involving Control-IQ from our review are compiled in *Table 3*.^13,15–17,24–27^

**Table 1:** Clinical and real-world studies involving Medtronic's MiniMed 670G^3–8^

Study	System	Study design	Sample	TIR (70–180mg/dL) (baseline, final, change, p-value)	HbA1c (baseline, final, change, p-value)	Time below range <70mg/dL (baseline, final, change, p-value)	Time above range >180mg/dL (baseline, final, change, p-value)	Adverse events
Garg et al. (2017)^3^	MiniMed^™^ 670G (Medtronic, Dublin, Ireland)	Three-month multicentre single-arm prospective clinical study	Adults with T1DM (n=94; age range: 22–75 years); adolescents with T1DM (n=30; age range: 14–21 yrs)	Adults: 68.8%; adolescents: 60.4%	Adults: 7.3%; adolescents: 7.7%	Adults: 6.4%; adolescents: 4.3%	Adults: 24.8% adolescents: 35.3%	No DKA or severe hypoglycaemia reported in either cohort
				Adults: 73.8%; adolescents: 67.2%	Adults: 6.8%; adolescents: 7.1%	Adults: 3.4%; adolescents: 2.8%	Adults: 22.8%; adolescents: 30.0%	
				Adults: +5.0% (+1.2 hrs/day); adolescents: +6.8% (+1.7 hrs/day)	Adults: -0.5%; adolescents: -0.6%	Adults: -3.0% (-43 mins/day); adolescents: -1.5% (-22 mins/day)	Adults: -2.0% (-29 mins/day); adolescents: -5.3% (-1.3 hrs/day)	
				Adults: p<0.001; adolescents: p<0.001	Adults: p<0.001; adolescents: p<0.001	Adults: p<0.001; adolescents: p<0.001	Adults: p= 0.01045 adolescents: p<0.001	
Forlenza et al. (2019)^4^	MiniMed 670G	Three-month multicentre single-arm prospective clinical study	Children with T1DM (N=105; age range: 7–13 yrs)	56.2%	7.9%	3.2%	40.6%	Severe hypoglycaemia: 0.89 events per 100 person-yrs during the run-in phase; 0.71 events per 100 person-yrs during the study phase. DKA: 1 episode during the whole trial; the patient withdrew
				65.0%	7.5%	1.2%	33.8%
				+8.8% (+2.2 hrs/day)	-0.4%	-2.0% (-29 mins/day)	-6.8% (-1.6 hrs/day)
				p<0.001	p<0.001	p=0.126	p<0.001
Bergenstal et al. (2016)^5^	MiniMed 670G	Three-month multicentre single-arm prospective clinical study	People with T1DM (N=124; age range: 14–75 yrs)	66.7%	7.4%	5.9%	27.4%	No severe hypoglycaemic or DKA events were detected. A total of 28 adverse events, including hyperglycaemia, were found
				72.2%	6.9%	3.3%	24.5%	
				+5.5% (+1.2 hrs/day)	-0.5%	-2.6% (-37 mins/day)	-2.9% (-42 mins/day)	
				N/A	N/A	N/A	N/A	
Akturk et al. (2019)^6^	MiniMed 670G	Six-month retrospective study	Adults with T1DM (N=127; age range: 21–68 yrs)	59.5%	7.6%	3.2%	37.3%	N/A
				70.1%	7.2%	2.2%	27.7%	
				+10.6% (+2.4 hrs/day)	-0.4%	-1.0% (-14 mins/day)	-9.6% (-2.3 hrs/day)	
				p<0.001	p<0.001	p<0.05	p<0.001	
Bergenstal et al.(2021)^7^	MiniMed 670G and MiniMed^™^ 780G (Medtronic, Dublin, Ireland)	Multicentre randomized crossover trial consisting of two 12-week periods, with Medtronic's MiniMed 670G system used during one 12-week period and MiniMed 780G system used during the other	People with T1DM (N=113; age range: 14–29 yrs)	Overall: 57.0%	Overall: 7.9%	Overall: 2.3%	Overall: 40.7%	One severe hypoglycaemic event in the 780G arm; 12 others for hyperglycaemia and 0 in the 670G arm. In total, Seven adverse events were reported for seven (6%) of 112 participants during in the 670G arm and six events for six (5%) of participants in the 780G arm
				670G: 63.0%; 780G: 67.0%	670G: 7.6%; 780G: 7.4%	Overall: 2.1%	670G: 34.9%; 780G: 30.9%	
				670G: +6.0% (+1.4 hrs/day); 780G: +10.0% (+2.4 hrs/day)	670G: -0.3%; 780G: -0.5%	Overall: -0.2% (-3 mins/day)	670G: -5.8% (-1.4 hrs/day); 780G: -9.8% (-2.4 hrs/day)	
				670G: p<0.001; 780G: p<0.001	670G: p<0.001; 780G: p<0.001	N/A	N/A	
Stone et al. (2018)^8^	MiniMed 670G	Three-month real-world study	People with T1DM (N=3,141; age range: 14–75 yrs)	66.0%	N/A	2.7%	31.4%	N/A
				73.3%	N/A	2.1%	24.6%	
				+7.0% (+1.7 hrs/day)	N/A	-0.6% (-9 mins/day)	-6.8% (-1.6 hrs/day)	

*DKA = diabetic ketoacidosis; HbA1c = glycated haemoglobin; hrs = hours; mins = minutes; N/A = not applicable; T1DM = type 1 diabetes; TIR = time in range; yr = year.*

**Table 2:** Clinical and real-world studies involving Medtronic's MiniMed 780G^9–12^

Study	System	Study design	Sample	TIR (baseline, final, change, p-value)	HbA1c (baseline, final, change, p-value)	Time below range (baseline, final, change, p-value)	Time above range (baseline, final, change, p-value)	Adverse events
Carlson et al. (2021)^9^	MiniMed^™^ 780G (Medtronic, Dublin, Ireland)	Single-arm, multicentre prospective clinical study	People with T1DM (N=157; age range: 14–75 yrs)	68.8%	7.5%	3.3%	27.9%	Three serious adverse events, all unrelated to the system: one severe hypoglycaemic event during run-in, one case of appendicitis and one of sepsis
				74.5%	7.0%	2.3%	23.1%	
				+5.7% (+1.4 hrs/day)	-0.5%	-1.0% (+14 mins/day)	-4.8% (-1.1 hrs/day)	
				p<0.001	p<0.001	p<0.001	p<0.001	
Collyns et al. (2021)^10^	MiniMed 780G	Dual centre randomized crossover trial consisting of two 4-week periods, with Medtronic's MiniMed 780G (closed-loop) system during one 4-week period and SAP + PLGM therapy during the other	People with T1DM (N=59; age range: 7–80 yrs)	Overall: 59.0%	Overall: 7.6%	Overall: 3.1%	Overall: 37.9%	A total of 5 of the 37 adverse events were related to the device; all were skin reactions
				PLGM: 57.9%; closed-loop: 70.4%	N/A	PLGM: 2.5%; closed-loop: 2.1%	PLGM: 39.6%; closed-loop: 27.5%	
				PLGM: -1.1% (-14 mins/day); closed-loop: +11.4% (+2.6 hrs/day)	N/A	PLGM: -0.6% (-9 mins/day); closed-loop: -1.0% (+14 mins/day)	PLGM: +1.7% (+24 mins/day); closed-loop: -10.4% (-2.5 hrs/day)	
				PLGM: p<0.001; closed-loop: p<0.001	N/A	PLGM: p<0.001; closed-loop: p<0.001	PLGM: p<0.001; closed-loop: p<0.001	
Nimri et al. (2021)^11^	MiniMed 780G	Single-arm prospective clinical study	Adolescents and adults with T1DM (N=12; median age: 17 yrs)	68.4%	7.1%	4.0%	27.6%	No serious adverse events occurred
				74.0%	6.8%	2.6%	23.4%	
				+5.6% (+1.4 hrs/day)	-0.3%	-1.4% (-20 mins/day)	-4.2% (-1.0 hrs/day)	
				p=0.06	p=0.0027	p=0.27	N/A	
Beato-Víbora et al. (2021)^12^	MiniMed 780G	Single-arm prospective clinical study	Participants with T1DM (N=52; mean age: 43 yrs)	67.3%	7.2%	N/A	N/A	N/A
				79.6%	N/A	N/A	N/A	
				+12.3% (+3.1 hrs/day)	N/A	No differences found	No differences found	
				p=0.001	N/A	N/A	N/A	

*HbA1c = glycated haemoglobin; hrs = hours; mins = minutes; N/A = not applicable; PLGM = predictive low glucose management; SAP = sensor-augmented pump; T1DM = type 1 diabetes; TIR = time in range; yrs = years.*

### Insulet's Omnipod 5

The Omnipod^®^ 5 (Insulet Corporation, Acton, MA, USA) AID system is considerably different from traditional pumps and, since being cleared by the FDA in January 2022, has marked the first and only wearable, tubeless system on the market.^16^ It comprises the tubeless Omnipod insulin patch pump, a Dexcom G6 CGM and a model predictive control algorithm. The patch pump can be worn for up to 3 days and can be fully controlled with an Android phone. In the USA, Insulet's unique business model provides coverage through pharmacy benefits, meaning that users can pay as they go. This model contrasts with traditional insulin pumps, which go through durable medical equipment suppliers and typically require a four-year contract. The one-piece tubeless pump is seen as an easier option for initiating patients than traditional pumps, which require a long-term contract and tubed infusion sites.^17^ Many people prefer a tubeless pump over a conventional pump with tubing. Prior to the Omnipod 5, the Omnipod Dash Omnipod DASH^®^ (Insulet Corporation, Acton, MA, USA) was available as a tubeless patch pump, however, it was not directly integrated with CGM.^28^

During the system's pivotal trial, 128 adult participants (age range: 14–71 years) and 112 paediatric participants (age range: 6–13.9 years) wore the Omnipod 5 AID system for 3 months, witnessing an improvement in TIR of 9.2% (2.1 hours) and 15.5% (3.9 hours) (from 64.7% to 73.9%, and from 52.5% to 68.0%), respectively.^26^ The mean HbA1c reduction was 0.38% in adults (from 7.16% to 6.78%) and 0.68% in children (from 7.67% to 6.99%). Time spent <70 mg/dL improved by 0.91% (13 minutes), bringing the adult arm to 1.1%; hypoglycaemia data were not collected for children. Similar glycaemic improvements were witnessed during the system's initial safety trial, during which 18 adults and 18 children saw an improvement of 6.9% and 13.9% in TIR, respectively, while not experiencing any hypoglycaemic or diabetic ketoacidosis events.^27^ All studies involving Omnipod 5 from our review are compiled in *Table 4*.^29,30^

### DIY systems

The do it yourself (DIY) movement of AID began in 2013 with a group of patients and their families who had become impatient with the slow pace of regulatory approval for AID systems. They met virtually under the hashtag #WeAreNotWaiting, and honed and shared open-source hardware and software. These groups were led by programmers willing to share code, others willing to test the code and users providing feedback for future software upgrades. Currently, there are three types of DIY options: OpenAPS, AndroidAPS and Loop.^31^

**Table 3:** Clinical and real-world studies involving Tandem's Control-IQ (continued)^13,15,24–27^

Study	System	Study design	Sample	TIR (baseline, final, change, p-value)	HbA1c (baseline, final, change, p-value)	Time below range (baseline, final, change, p-value)	Time above range (baseline, final, change, p-value)	Adverse events
Brown et al. (2019)^13^	Control-IQ Technology^™^ (Tandem Diabetes Care, Inc., San Diego, CA, USA)	Six-month multicentre randomized controlled trial of Control-IQ (closed-loop) versus SAP therapy (t:slim X2 pump and G6 in open loop)	People with T1DM (N=168; age range: 14–71 yrs)	Closed-loop: 61.0%; SAP: 59.0%	Closed-loop: 7.4%; SAP: 7.4%	Closed-loop: 3.6%; SAP: 2.9%	Closed-loop: 35.4%; SAP: 38.1%	Adverse events included DKA, hyperglycaemia/ketosis and hospitalization. The closed-loop cohort (n=112) had 30.2 adverse events per 100 person-yrs, SAP control group (n=56) had 7.1 adverse events per 100 person-yrs (p=0.05)
				Closed-loop: 71.0%; SAP: 59.0%	Closed-loop: 7.06%; SAP: 7.4%	Closed-loop: 1.6%; SAP: 2.3%	Closed-loop: 27.4%; SAP: 38.7%	
				Closed-loop: +10.0% (+2.4 hrs/day); SAP: 0.0%	Closed-loop: -0.3%; SAP: 0.0%	Closed-loop: -2.0% (-29 mins/day); SAP: -0.6% (-9 mins/day)	Closed-loop: -8.0% (-1.9 hrs/day); SAP: +0.6% (+9 mins/day)	
				Closed-loop: p<0.001; SAP: p<0.001	Closed-loop: p=0.001; SAP: p=0.001	Closed-loop: p<0.001; SAP: p<0.001	Closed-loop: p<0.001; SAP: p<0.001	
Breton et al. (2020)^15^	Control-IQ	Four-month multicentre randomized controlled trial of at-home use of Control-IQ versus sensor-augmented pump therapy (t:slim X2 pump and Dexcom G6 CGM)	Children with T1DM (N=101;age range: 6–13 yrs)	Closed-loop: 53.0%; SAP: 51.0%	Closed-loop: 7.6%, SAP: 7.9%	Closed-loop: 1.2%; SAP: 1.0%	Closed-loop: 45.8%; SAP: 48%	Included hyperglycaemia/ketosis. Closed-loop cohort (n=78) had 65.3 adverse events per 100 person-yrs, The SAP control group (n=23) had 41.3 adverse events per 100 person-yrs (p=0.50)
				Closed-loop: 67.0%; SAP: 55.0%	Closed-loop: 7.0%; SAP: 7.6%	Closed-loop: 1.6%; SAP: 1.8%	Closed-loop: 31.4%; SAP: 43.2%	
				Closed-loop: +14.0% (+3.4 hrs/day); SAP: +4.0% (+1 hrs/day)	Closed-loop: -0.6% ; SAP: -0.3%	Closed-loop: -0.4% (-6 min/day); SAP: +0.8% (+12 mins/day)	Closed-loop: -14.4% (-3.5 hrs/day); SAP: -4.8% (-1.1 hrs/day)	
				Closed-loop: p<0.001; SAP: p<0.001	Closed-loop: p<0.001; SAP: p<0.001	Closed-loop: p<0.001; SAP: p<0.001	Closed-loop: p<0.001; SAP: p<0.001	
Forlenza et al. (2019)^25^	Control-IQ	Three-day multicentre randomized controlled trial of at-home use of Control-IQ versus sensor-augmented pump therapy (t:slim X2 pump and Dexcom G6 CGM)	Children with T1DM (N=24; age range: 6–12 yrs)	N/A	Overall: 7.4%	N/A	N/A	Adverse events included DKA (2 participants) and hypoglycaemia (2 participants)
				Closed-loop: 71.0%; SAP: 52.8%	N/A	Closed-loop: 2.1%; SAP: 2.1%	Closed-loop: 26.9%; SAP: 45.1%	
				N/A	N/A	N/A	N/A	
				Between arms: p<0.001	N/A	Between arms: p>0.05 (not significant)	Between arms: p<0.001	
Ekhlaspour et al. (2021)^26^	Control-IQ	Three-month multicentre single-arm prospective clinical study	Children with T1DM (N=12;age range: 2–5 yrs)	61.7%	7.3%	3.7%	34.1%	All participants completed the study with no adverse events
				71.3%	N/A	3.2%	25.7%	
				+9.6% (+2.3 hrs/day)	N/A	-0.5% (-7 mins/day)	-8.4% (-2.0 hrs/day)	
				p=0.016	N/A	p=0.182	p=0.042	
Forlenza et al. (2018)^27^	Basal-IQ^®^ Technology (Tandem Diabetes Care, Inc., San Diego, CA, USA)	Multicentre randomized crossover trial consisting of two 3-week periods with Basal-IQ (PLGS) used during one 3-week period, and SAP therapy (t:slim X2 pump and Dexcom G5 CGM with PLGS system disabled) used during the other	People with T1DM (N=102; age range: 6–72 yrs)	Overall: 64.0%	Overall: 7.3%	Overall: 3.6%	Overall: 32.4%	One severe hypoglycaemic event occurred during the SAP arm
				PLGS: 65.0%; SAP: 63.0%	N/A	PLGS: 2.6%; SAP: 3.2%	PLGS: 32.4%; SAP: 33.8%	
				PLGS: +1.0% (+14 mins/day); SAP: -2.0% (-29 mins/day)	N/A	PLGS: -1.0% (-14 mins/day); SAP: -0.4% (-6 mins/day)	PLGS: 0%; SAP: +1.4% (+20 mins/day)	
				p<0.001	N/A	p<0.001	p=0.12	
Breton et al. (2021)^24^	Control-IQ	Year-long real-world retrospective study	People with diabetes (N=9,451, 83% with T1DM; age range: 6–91 yrs)	63.6%	GMI: 7.3%	~1.0%	33.2%	N/A
				73.6%	N/A	~1.0%	24.3%	
				+10.0% (+2.4 hrs/day)	N/A	~0.0%	-8.9% (-2.1 hrs/day)	

*DKA = diabetic ketoacidosis; GMI = glucose management indicator; HbA1c = glycated haemoglobin; hrs = hours; mins = minutes; N/A = not applicable; PLGS = predictive low-glucose suspend; PLGM = predictive low glucose management; SAP = sensor-augmented pump; T1DM = type 1 diabetes; TIR = time in range; yrs = years.*

The DIY code is shared online, and patients, family members or support persons can download tested code to build their own DIY system. Patients can set their own pump settings, including target blood glucose, basal rates and sensitivity factors, and initiate the system using specific older versions of Medtronic insulin pumps, the original Omnipod Eros or other compatible insulin pumps (e.g. DANA Diabecare R and RS insulin pumps, and the Roche Accu-Chek Insight insulin pump). Support is found through online platforms such as Facebook (Meta Platforms, Inc., Menlo Park, CA, USA), Twitter (Twitter, San Francisco, CA, USA) and NightScout.^32^ Manufacturers do not directly offer support for these systems.

There are no randomized controlled studies on DIY system use at the time of writing. In a systematic review, 6 observational studies, 2 case reports and 1 anecdotal study were found.^33^ Benefits, such as improved quality of life, reduced fear of hypoglycaemia and improved TIR, are noted across these studies; however, these studies are all small, self-reported and with an observational or retrospective design. The CREATE (Community deRivEd AutomaTEd insulin delivery) trial (Randomised parallel arm open label clinical trial comparing automated insulin delivery using an open-source algorithm [AnyDANA-loop], with sensor augmented pump therapy in type 1 diabetes; Registration number: ACTRN12620000034932), a randomized parallel arm open-label clinical trial conducted over 24 weeks, compared the AID system using a mobile controller (AnyDANA-loop) with an open-source algorithm with SAP therapy in participants with T1DM recruited from 4 sites in New Zealand.^34,35^ Most recently, Lum et al. published a paper in *Diabetes Technology & Therapeutics* showing that the open-source Loop AID system can safely and effectively be initiated in adults and children with T1DM via community-developed resources.^36^ In this prospective real-world observational study, the mean TIR of both adult and paediatric participants improved by 1.6 hours/day to 73%.

## Automated insulin delivery and personalized care: How to support patients

Supporting patients who choose to use AID systems will become more necessary as systems gain a wider audience of users and other AID systems come to market. In a study of MiniMed 670G users, 30% of youths who discontinued AID use stated difficulty with alarms, the number of calibrations to keep the system functioning and the time needed to keep the system functioning as key reasons for discontinuation, with higher HbA1c noted as a predictor of discontinuation.^35^ Newer AID models require fewer to no calibrations and less intensive interaction. Discussions should be started before selecting a given AID system, and health care professionals must ensure that patients know that all current manufacturers require manual bolus doses for food and that there is currently no system that is completely free of patient interaction.^37^

**Table 4:** Clinical and real-world studies involving Omnipod 5^26,27^

Study	System	Study design	Sample	TIR (baseline, final, change, p-value)	HbA1c (baseline, final, change, p-value)	Time below range (baseline, final, change, p-value)	Time above range (baseline, final, change, p-value)	Adverse events
Brown et al. (2021)^26^	Omnipod^®^5 (Insulet Corporation, Acton, MA, USA)	Three-month multicentre single-arm prospective clinical study	Adults with T1DM (N=128; age range: 17–70 yrs); children with T1DM (N=112;age range; 6–13.9 yrs)	Adults: 64.7%; children: 52.5%	Adults: 7.16%; children: 7.67%	Adults: 2.89% children: 2.21%	Adults: 32.41% children: 45.29%	Severe hypoglycaemia: a total of 4.8 events per 100 person-yrs (adults: 6.0 events/100 person-yrs; children: 3.6 events/100 person-yrs); DKA: a total of 1.2 events per 100 person-yrs (adults: 0.0 events/100 person-yrs; children: 3.6 events/100 person-yrs)
				Adults: 73.9%; children: 68.0%	Adults: 6.78%; children: 6.99%	Adults: 1.32% children: 1.78%	Adults: 24.78% children: 30.22%	
				Adults: +9.3% (+2.2 hrs/day); children: +15.6% (+3.8 hrs/day)	Adults: -0.38%; children: -0.71%	Adults: -1.57% (-23 mins/day) children: -0.43% (-6 mins/day)	Adults: -7.63% (-1.8 hrs/day) children: -15.07% (-3.6 hrs/day)	
				Adults: p<0.0001; children: p<0.0001	Adults: p<0.0001; children: p<0.0001	Adults: p<0.0001 children: p=0.8153	Adults: p<0.0001; children: p<0.0001	
Forlenza et al. (2021)^27^	Omnipod 5	Single-arm multicentre prospective clinical trial	Adults with T1DM (N=18; age range: 14–70 yrs); children with T1DM (N=18; age range; 6–13.9 yrs)	Adults: 65.6%; children: 51.0%	Adults: 7.1%; children: 7.8%	Adults: 3.4%; children: 2.3%	Adults: 30.9%; children: 46.7%	No hypoglycaemia or DKA; 1 paediatric adverse event and 3 adult adverse events, 1 of which was prolonged hyperglycaemia, were found
				Adults: 72.5%; children: 64.9%	N/A	Adults: 0.7%; children: 1.1%	Adults: 26.8%; children: 34%	
				Adults: +7% (+1.7 hrs/day); children: +13.9% (+3.3 hrs/day)	N/A	Adults: -2.7% (-39 mins/day); children: -1.2% (-17 mins/day)	Adults: -4.1% (-59 mins/day); children: -12.7% (-3.0 hrs/day)	
				Adults: N/A; children: p<0.01	N/A	Adults: p<0.01; children: p<0.05	Adults: N/A; children: p<0.01	

*DKA = diabetic ketoacidosis; HbA1c = glycated haemoglobin; hrs = hours; mins = minutes; N/A = not applicable; T1DM = type 1 diabetes; TIR = time in range; yrs = years.*

The CARES framework (Calculate, Adjust, Revert, Educate, Sensor/Share) can be used with patients to discuss how a system works when dosing insulin.^38^ In particular, the use of this paradigm within patient interactions involves discussing “how each system *calculates* insulin delivery, which parameters can be *adjusted*, when users should *revert* to traditional insulin pump settings, critical education points, and key aspects of the *sensor* and *sharing* capabilities”.^38^

During patient encounters, providers can benefit from downloading standardized reports, such as the Ambulatory Glucose Profile and AID product-specific reports, to open discussions on the timing of mealtime insulin, the treatment of hyperglycaemia and hypoglycaemia, the use of overrides (e.g. exercise), and when to increase target blood glucose.^39^ In keeping with the International TIR Guidelines, dialogue on CGM-derived metrics (TIR, time below range and time above range) and improvements or challenges since the last patient–clinician interaction can improve glycaemic outcomes, help with goal setting and guide the timing of follow-up appointments.^40^

The Identify, Collaborate, Configure framework can help optimize technology-enabled diabetes management by identifying the right technology at the right time for each patient, knowing this may change over time as patient circumstances and disease states change.^41^ Configuring devices with goals and targets that are patient specific, incorporating them into the treatment plan, and providing on-going support for questions, changes in health status and updates in technology will enable patients to use their technology to manage their disease sustainably. Collaborating with patients and engaging in data-driven conversations with shared decision-making and integrating the entire care team optimizes patient outcomes.

## The future of automated insulin delivery

As noted above, AID systems cannot meet the needs of all people with T1DM, and multiple daily injections will remain an attractive option for many in the near future. Future iterations of AID systems will need to “close the loop” and remove the need for exercise announcements or meal boluses. One such example is the bihormonal iLet^®^ Bionic Pancreas (Beta Bionics^®^, Irvine, CA, USA), a dual hormone pump that uses glucagon and insulin to regulate an individual's blood glucose level. During an open-label random-order crossover trial, 10 participants used the system and experienced statistically and clinically significant improvements while using this system, compared with the insulin-only configuration of the Bionic Pancreas.^42^ While this technology is a promising step towards the “set it and forget it” goal of AID, it is far from receiving FDA approval. One of the other challenges to this goal is the speed at which insulin works. There have been strides toward faster insulin pumps, such as insulin lispro-aabc and the faster-acting insulin aspart. However, these insulins still require user-initiated boluses in advance of eating.

In the meantime, it is important to find material ways to improve AID for patients through increased education at the provider and patient levels. Besides improvements in insurance coverage and device interoperability, there must be an increased awareness of how to customize AID system settings to meet the unique needs of each patient, for example, via tip sheets or customized glucose targets during different points of the day; furthermore, systematic approaches are needed for providers to understand which system may be most appropriate for which patient. On-going and future studies must also show the glycaemic benefits of AID use in specific groups, for example, in geriatric or pregnant populations with T1DM or those with new-onset T1DM for beta cell preservation.

## Conclusions

AID has come a long way, starting with systems that could only suspend insulin delivery when glucose was low and evolving into systems that can suspend insulin delivery if glucose is predicted to go low. Now, we have systems that can increase or decrease insulin based on predicted glucose values and give automated correction bolus doses as needed. There is plenty of evidence that supports the use of these systems as a standard of care for people with T1DM, as evidenced by the improvements in TIR and other glycaemic and patient-reported outcomes. Understanding the differences between each system and ensuring proper patient and provider education is important to ensure optimal outcomes. While many have used AID and the term “artificial pancreas” interchangeably for some time, we argue that there is no true artificial pancreas as of now, given that users must still initiate meal boluses and engage with the system to ensure consistent glycaemic management. The future does look very bright for AID systems, which will likely become even more automated by adding glucagon and incorporating faster insulins. These improvements may ultimately reduce the need for mealtime bolus insulin doses or additional carbohydrate intake for exercise.
